# Association between migration status and subsequent labour market marginalisation among individuals with posttraumatic stress disorder: a Swedish nationwide register-based cohort study

**DOI:** 10.1007/s00127-022-02263-5

**Published:** 2022-03-21

**Authors:** Jiangchuan He, Anna-Clara Hollander, Syed Rahman

**Affiliations:** Epidemiology of Psychiatric Conditions, Substance Use and Social Environment (EPiCSS), Department of Global Public Health, Karolinska Institutet (KI), Solnavägen 1E, 113 65 Stockholm, Sweden

**Keywords:** Migration, Posttraumatic stress disorder, Labour market marginalisation, Unemployment, Sickness absence, Disability pension

## Abstract

**Purpose:**

The high prevalence of posttraumatic stress disorder (PTSD) among migrants in Europe is widely reported. Our research aimed to investigate the association between migration status and subsequent labour market marginalisation (LMM) events, i.e., long-term unemployment (LTU), long-term sickness absence (LTSA), and disability pension (DP) among individuals with PTSD, and to elucidate how the sociodemographic factors and the pre-existing health conditions influence such association.

**Methods:**

We established a cohort of 36,714 individuals born between 1960 and 1995, living in Sweden during 2004–2009, aged 19 years or above, with PTSD diagnosis during 2006–2009. Migration status, categorized as refugees, non-refugees, second-generation migrants, and Swedish-born with Swedish-born parents (reference group) was considered as exposure and LMM events as outcome. The cohort was followed from 01-Jan-2010 until LMM, death, or end of follow-up (31-Dec-2016). Hazard ratios (HRs) with 95% confidence intervals (CIs) were estimated by Cox regression with a seven-year follow-up.

**Results:**

Refugees (HR 2.07, 95% CI 1.86–2.30), and non-refugees (HR 1.96, 95% CI 1.85–2.07) had almost doubled relative risk of long-term unemployment, compared with the Swedish-born. The hazards of long-term sickness absence were similar across the groups. Refugees (HR 1.49, 95% CI 1.24–1.77), and non-refugees (HR 1.42, 95% CI 1.30–1.56) also had elevated relative risk of disability pension, whereas second-generation migrants had moderately increased relative risks for all three labour market marginalisation events compared with the Swedish-born.

**Conclusion:**

Among the individuals with PTSD, being a migrant increases the risk of LMM, refugees being the foremost among migrants. Further research may benefit from including more recent migrant population, pre-migration information and measuring PTSD clinical severity.

**Supplementary Information:**

The online version contains supplementary material available at 10.1007/s00127-022-02263-5.

## Background

Worldwide, approximately 70% of the adult population reported having been exposed to a traumatic event at least once in their lifetime [1–3]. Some of the traumatized persons will eventually develop posttraumatic stress disorder (PTSD). The symptoms of PTSD include severe anxiety, persistent avoidance, intrusive thoughts, etc., therefore PTSD could easily interfere with ones’ social and occupational life [4, 5]. According to the World Mental Health Survey, the lifetime prevalence of PTSD among adults was 6.9% in the U.S., while a lower average rate (1.9%) was observed among six Western European countries (i.e., Belgium, France, Germany, Italy, the Netherlands, and Spain) [6, 7]. In Sweden, the lifetime PTSD prevalence among the adult population has been reported to be 5.6%, which is much higher than in other EU-countries [8]. Worth mentioning that the PTSD prevalence are usually derived from survey data, therefore, inclined to different biases and could also be a potential reason for such wide differences in PTSD prevalence between the countries.

During the last decade, a considerable influx of migrants has been recorded throughout Europe each year, and the number is growing. In the year 2010, 20.1 million (4.9%) out of the 440.7 million people living in the EU-27 countries were non-EU-27 citizens [9, 10]. Sweden, relative to its population size, is one of the European countries which have received the greatest number of migrants, as from 2010 to 2019, more than one million people in total have migrated to Sweden [11]. In 2010, over 14.76% (1.38 million) of the Swedish population were foreign-born, of which 12% were refugees [11, 12].

Existing studies have identified an elevated risk of mental disorders including PTSD, among migrants and particularly in refugees, compared with the host population [13–16]. In Sweden, foreign-born individuals reported traumatic experiences more frequently, and they had an almost threefold higher lifetime prevalence of PTSD than their Swedish-born peers (12.9% vs 4.6%) [8, 16]. A recent Swedish study, using the national patient register to compare somatic and mental disorders regarding subsequent disability pension and mortality, reported prevalence of in- or specialised outpatient healthcare use due to PTSD to be 2.0 and 0.1% among refugees and Swedish host population, respectively [17].

It is essential for adult migrants to become a part of the labour force, not only to acquire income but also to integrate into the host society [18, 19]. In Sweden, migrants make up to 10% of the total working force, nevertheless the employment rate among migrants remains less than 60% even after ten years of resettlement [20, 21]. Additionally, refugee migrants are found more likely to be marginalised from the labour market, that is, temporarily or permanently unable to join (i.e., unemployment) or maintain in the labour force (i.e., sickness absence and disability pension) [22].

Several studies have looked at the risk differences between refugees and Swedes among the young population with common mental disorders (CMDs) [23–25], as unlike severe mental disorders, such as schizophrenia or other psychotic disorders [26, 27], people with PTSD or other CMDs have a great potential to engage into the labour market if timely and adequate measures are taken. Nevertheless, the research so far has focused on CMDs in general, not separating effects of specific disorders, i.e., depression, anxiety or stress-related mental disorders. In addition, it is not clear if the same mental disorder poses the same risk of labour market marginalisation among those born in their own country, migrants and their children. In some of the previous studies, the sample sizes were rather small, and the second-generation migrants were often merged into the native-born category [24, 28, 29]. However, it has been acknowledged that second-generation migrants have their specific risk, not shared with the first-generation migrants nor with the domestic-born [30]. Given the great proportion of migrants living in Europe including Sweden, the elevated risks of PTSD and the low employment rate among migrants, it is crucial to examine the factors facilitating to join and remain in the labour market for migrants with PTSD. To date, no study has dealt with this issue. Our study is not only unique in examining the individuals with PTSD at their working age, a disorder that is much more prevalent in refugees than in the host population, but also in discriminating migrants with and without refugee experience and separating second generation migrants. This study aimed to investigate the association between migration status and subsequent labour market marginalisation among individuals with PTSD. A further aim was to elucidate how the sociodemographic factors and pre-existing health conditions influence such association.

## Method

### Data sources

This prospective cohort study was based on a register linkage called Psychiatry Sweden (https://ki.se/en/gph/psychiatry-sweden-the-register-linkage-epicss-group) [31]. It is an anonymized database of multiple Swedish nationwide registers including data from Statistics Sweden, the Social Insurance Agency, and the National Board of Health and Welfare. Each person’s sociodemographic characteristics and health-related records were linked at an individual level by applying the anonymized personal number, which is unique for all Swedish residents.

### Population

All individuals born between 1960–1995, registered and living in Sweden between 2004 and 2009, aged 19 or above on 31-Dec-2009 were included in the study base (*N* = 4,315,485), excluding temporary visitors and those without a residency permit, such as asylum seekers and undocumented migrants. Additional inclusion criterion was PTSD diagnosis. From the study base, only those with inpatient or specialised outpatient healthcare use due to PTSD-diagnose (ICD 10: F43.1) between 2006 and 2009 were included, while to enrol recent PTSD cases only, the cases earlier than 2006 were ruled out (*n* = 15,075). Additionally, individuals with comorbid psychotic disorders (ICD 10: F20-31) were excluded at the baseline, given the excessive-high risks of labour market marginalisation in this group have been reported [26, 27]. Those who emigrated (*n* = 73,365), died (*n* = 13,020), or had ongoing disability pension (*n* = 2) or old-age pension (*n* = 2) before 01-Jan-2010 were excluded. The final study population consisted of 36,714 individuals and was followed from 01-Jan-2010 to 31-Dec-2016.

### Outcome measurement

The study conceptualized labour market marginalisation by three separate outcomes: (i) long-term unemployment, (ii) long-term sickness absence, and iii) disability pension. Regarding unemployment: in Sweden, all persons aged 16 years and above who acquire income from work are eligible to receive either basic or income-related unemployment benefit once they get registered as unemployed [20, 32]. Sickness absence, also known as sick leave, refers to the temporarily reduced work capacity due to disease or injury. A person aged 16 years or older on sickness absence receives compensation from the employer from day 2 to day 14, while the Swedish Social Insurance Agency pays sickness benefit from day 15 and onwards. The first day of a sick leave spell is considered as a qualifying day, which is not compensated financially [33]. Disability pension is a form of public financial support provided to the individuals who permanently leave the labour market before the age of statutory retirement due to disease or injury, which is usually preceded by sick leave [33].

In this study, ‘long-term unemployment’ refers to being unemployed for more than 180 days in a calendar year, and ‘long-term sickness absence’ implies to anyone who is on sick leave for more than 90 net days in a year. “Net day” was defined as the duration of being entitled to the sickness absence multiplied by the extent of absence (25%, 50%, 75% and 100%). For instance, ten days of sickness absence with a 50% absence extent is counted as five net days. Similar definitions of long-term unemployment and long-term sickness absence have been used previously by other researchers from Sweden [17, 23–25]. Disability pension, as well as the other two outcomes were defined as dichotomous measurements with possibilities ‘yes/no’. All information regarding labour market marginalisation was collected from Statistics Sweden’s Longitudinal Integrated Database for Health Insurance and Labour Market Studies (LISA) [34].

### Exposure variables

Migration status, the exposure, was divided into four categories, namely, Swedish-born with Swedish born parents (from now on Swedish), refugee migrants (from now on refugees), first-generation non-refugee migrants (from now on non-refugees), and second-generation migrants. Refugees were defined according to the Swedish Migration Agency’s definition of refugee status, made in accordance with Swedish law and the UN Refugee Convention, as someone who, “owing to a well-founded fear of being persecuted... is unable to, or owing to such fear, is unwilling to avail himself of the protection of that country [35].” Non-refugees are those who were born abroad with both parents born abroad and later settled in Sweden for work, family reunion, study, investments, etc. Second-generation migrants were defined as born in Sweden with at least one parent born abroad. The exposure status was measured by retrieving information from Statistics Sweden’s longitudinal database for integration studies (STATIV) [36] and multi-generation register [37].

### Covariates

All covariates were selected based on the existing scientific knowledge in regard to migration, mental health, and labour market. Sociodemographic factors were retrieved from LISA, which included age, sex, emigration, weighted household disposable income, and family composition. All socioeconomic variables were measured at baseline, i.e., on 31-Dec-2009, the day preceding the start of follow-up.

The pre-existing health-related factors, such as existing morbidity recorded before the start of follow-up, were divided into (i) other common mental disorders than PTSD (ICD 10: F32, F33, F40–43, except F43.1); (ii) other mental disorders (ICD 10: F00-99, except for diagnoses above); iii) major somatic disorders including diabetes mellitus (ICD 10: E10–E14), diseases of the circulatory system (ICD 10: I00–I99), diseases of the respiratory system (ICD 10: J00–J99), diseases of the musculoskeletal system and connective tissue (ICD 10: M00–M99) (Supplementary Table S1). All information on the aforementioned diagnoses were obtained from the Swedish National Patient Register at the Swedish Board of Health and Welfare, where the dates of admission were collected, and healthcare diagnoses were dichotomised as ‘yes/ no’. Information on health-related factors were obtained during the 4-year-window before the start of follow-up, precisely between 01-Jan-2006 and 31-Dec-2009.

### Statistical analysis

Hazard ratios (HRs) and 95% confidence intervals (CIs) regarding the three considered outcome measures of labour market marginalisation (i.e., long-term unemployment, long-term sickness absence, and disability pension) were estimated separately, with the Swedish as the reference group. Multivariable Cox proportional hazard models were applied, given the proportional hazard assumption was met. Kaplan–Meier’s curves were used for all three outcomes separately and visually checked to ensure the proportional hazard assumption. Censoring was applied to death, emigration, or the end of follow-up (31-Dec-2016) whichever occurred first. Furthermore, disability pension was presumed as permanent impairment of working capacity and was thereby considered a censoring event for analyses regarding long-term unemployment and long-term sickness absence. Similarly, long-term sickness absence was considered as an additional censoring event for the analysis of long-term unemployment as an outcome. All regression analyses were initially adjusted for sociodemographic factors (i.e., sex, age, weighted household disposable income, and family composition), and additionally adjusted for health-related factors (i.e., pre-existing major somatic disorders, pre-existing other common mental disorders, and pre-existing other mental disorders). Only covariates that seemed statistically significant from the crude model were included in the further adjustments. All statistical analyses were performed by STATA/IC 15.1 statistical package.

## Results

The study population consisted of 24 445 (66.6%) Swedish, 1023 (2.8%) refugees, 6376 (17.4%) non-refugee migrants, and 4870 (13.3%) second-generation migrants, all with PTSD diagnosed during 2006–2009. More females were found in each group except among the refugees, where sex was equally distributed (Table 1). First-generation migrants, i.e., refugees and non-refugees, were older (30+ years) compared with those born in Sweden, i.e., Swedish and second-generation migrants (83.9–86.1 vs 74.5–77.3%). Additionally, first-generation migrants to a greater extent lived at the lowest household disposable income level. Regarding family composition, Swedish and the second-generation migrants were found more likely to live alone (40.0–42.5%), while in comparison, more of the first-generation migrants were living with partner and children (41.5–43.1%). Prevalence of pre-existing major somatic disorders was similar across all groups, however, pre-existing mental disorders were more prevalent among the first-generation migrants (Table 1).

**Table 1:** Cohort characteristics by migration status among individuals born between 1960 and 1995, lived in Sweden as resident during 2004–2009, with post-traumatic stress disorder diagnosed between 2006–09

Characteristics	Swedish (*n* = 24,445)	Refugees (*n* = 1023)	Non-refugees (*n* = 6376)	2nd-generation migrants (*n* = 4870)
	No. (%)	No. (%)	No. (%)	No. (%)
Sociodemographic factors				
Sex				
Female	14,957 (61.2)	516 (50.4)	4156 (65.2)	2962 (60.8)
Male	9488 (38.8)	507 (49.6)	2220 (34.8)	1908 (39.2)
Age (years)				
19–29	5556 (22.7)	142 (13.9)	1025 (16.1)	1240 (25.5)
30 or more	18,889 (77.3)	881 (86.1)	5351 (83.9)	3630 (74.5)
Weighted household disposable income (in quintile)				
Lowest	4630 (18.9)	565 (55.2)	2977 (46.2)	1261 (25.9)
Second	5292 (21.6)	200 (19.6)	1216 (19.1)	1086 (22.3)
Third	5609 (22.9)	113 (11.0)	1018 (16.0)	951 (19.5)
Fourth	4958 (20.3)	97 (9.5)	700 (11.0)	835 (17.1)
Highest	3965 (16.2)	48 (4.7)	501 (7.9)	737 (15.1)
Family composition				
Living with partner without children	774 (3.2)	67 (6.5)	627 (9.8)	163 (3.3)
Living with partner and children	8314 (34.0)	441 (43.1)	2645 (41.5)	1364 (28.0)
Single without children	9780 (40.0)	335 (32.7)	2006 (31.5)	2069 (42.5)
Single with children	2083 (8.5)	116 (11.3)	726 (11.4)	485 (10.0)
Single age < 20, living with parents	3494 (14.3)	64 (6.3)	372 (5.8)	789 (16.2)
Pre-existing health condition^a, b^				
Major somatic disorders				
Yes	19,581 (80.1)	828 (80.9)	5147 (80.7)	3974 (81.6)
No	4864 (19.9)	195 (19.1)	1229 (19.3)	896 (18.4)
Other common mental disorders				
Yes	1350 (5.5)	88 (8.6)	494 (7.7)	259 (5.3)
No	23,095 (94.5)	935 (91.4)	5882 (92.3)	4611 (94.7)
Other mental disorders				
Yes	694 (2.8)	37 (3.6)	234 (3.7)	129 (2.6)
No	23,751 (97.2)	986 (96.4)	6142 (96.3)	4741 (97.4)
Labour market marginalisation event during the follow-up^c^				
Disability pension	951 (3.9)	72 (7.0)	300 (4.7)	235 (4.8)
Long-term unemployment	2177 (8.9)	286 (28.0)	1540 (24.2)	617 (12.7)
Long-term sickness absence	2870 (11.7)	114 (11.1)	678 (10.6)	616 (12.6)

All variables were measured at the end of 2009

^a^Pre-existing refers to before the start of follow-up (1-Jan-2010)

^b^For detailed disease classification please see Supplementary Table S1

^c^Follow-up period: 01-Jan-2010 to 31-Dec-2016

During the follow-up, 4620 (16.2%) individuals were long-term unemployed, commonly among the first-generation migrants compared with the Swedish and second-generation migrants (24.2–28.0 vs 8.9–12.7%). Regarding long-term sickness absence (*n* = 4278, 11.7%), the prevalence was similar across each group (10.6–12.6%). The share of disability pension, in general, was low (*n* = 1558, 4.2%) throughout the study, nevertheless was more prevalent among the refugees, compared with other groups (7.0 vs 3.9–4.8%) (Table 1).

### Long-term unemployment

Regarding the long-term unemployment, as compared with the Swedish, both refugees (HR 2.81, 95% CI 2.53–3.12) and the non-refugees (HR 2.42, 95% CI 2.29–2.55) had a nearly three times higher hazards in the crude model, meanwhile, second-generation migrants had only a moderate increased relative risk (HR 1.38, 95% CI 1.29–1.48). In the fully adjusted model (Model 3), where several sociodemographic and health-related factors were taken into account, the risk estimates for long-term unemployment considerably decreased but remained nearly two times higher especially among the first-generation migrants, i.e., refugees (HR 2.07, 95% CI 1.86–2.30) (Table 2) and non-refugees (HR 1.96, 95% CI 1.85–2.07).

**Table 2:** Crude and multivariable hazard ratios (HRs) with 95% confidence intervals (CIs) for *long-term unemployment* (> 180 days) among individuals born between 1960 and 1995, registered as living in Sweden in 2004–2009, age 19 years or older in 2010, with posttraumatic stress disorder diagnosed between 2006 and 2009

Characteristics	No. (%)	HR (95% CI) for long-term unemployment		
		Model 1^a^	Model 2^b^	Model 3^c^
Migration status				
Swedish	2 177 (8.9)	1.00	1.00	1.00
Refugee migrants	286 (28.0)	2.81 (2.53–3.12)	2.06 (1.86–2.30)	2.07 (1.86–2.30)
Non-refugee migrants	1 540 (24.2)	2.42 (2.29–2.55)	1.95 (1.84–2.06)	1.96 (1.85–2.07)
2nd-generation migrants	617 (12.7)	1.38 (1.29–1.48)	1.28 (1.19–1.37)	1.27 (1.18–1.36)

^a^Model 1: Crude analysis

^b^Model 2: Adjusted for age, sex, household disposable income, and family composition

^c^Model 3: Further adjusted for pre-existing major somatic disorders

Additional analyses revealed that males had 27% increased relative risk (HR 1.27, 95% CI 1.21–1.33) for subsequent long-term unemployment than females (Supplementary Table S2). Besides, a dose–response protective effect by household disposable income was observed, as individuals with a higher household disposable income were less likely to encounter long-term unemployment. Furthermore, those with pre-existing major somatic disorders were at an elevated relative risk for long-term unemployment (HR 1.40, 95% CI 1.32–1.50) regardless of their migration status, whereas other mental health conditions (except PTSD) had no such effect.

### Long-term sickness absence

The risk estimates for long-term sickness absence in comparison with the Swedish population were 1.30 (95% CI 1.14–1.48) for refugees, 1.21 (95% CI 1.14–1.28) for non-refugees, and 1.16 (95% CI 1.09–1.24) for second-generation migrants, respectively in the crude analyses. Adjustment for sociodemographic and health-related factors resulted in moderate decreases in risk estimates among each group (Table 3).

**Table 3:** Crude and multivariable hazard ratios (HRs) with 95% confidence intervals (CIs) for *long-term sickness absence* (> 90 net days) among individuals born between 1960 and 1995, registered as living in Sweden in 2004–2009, age 19 years or older in 2010, with posttraumatic stress disorder diagnosed between 2006 and 2009

Characteristics	No. (%)	HR (95% CI) for long-term sickness absence		
		Model 1^a^	Model 2^b^	Model 3^c^
Migration status				
Swedish	2 870 (11.7)	1.00	1.00	1.00
Refugee migrants	114 (11.1)	1.30 (1.14–1.48)	1.16 (1.02–1.33)	1.18 (1.03–1.35)
Non-refugee migrants	678 (10.6)	1.21 (1.14–1.28)	1.10 (1.03–1.17)	1.11 (1.04–1.19)
2nd-generation Migrants	616 (12.6)	1.16 (1.09–1.24)	1.14 (1.06–1.22)	1.13 (1.05–1.21)

^a^Model 1: crude analysis

^b^Model 2: adjusted for age, sex, household disposable income

^c^Model 3: further adjusted for pre-existing major somatic disorders, and other common mental disorders

In contrast to the other labour market marginalisation events, the relative risk for long-term sickness absence was slightly lower among males compared with females (HR 0.92, 95% CI 0.88–0.97) (Supplementary Table S3). In the full model, the risk decreased with increasing household disposable income. Pre-existing somatic comorbidity was found to increase the hazards of subsequent long-term sickness absence in individuals with PTSD by 60% (HR 1.60, 95% CI 1.49–1.71), whereas pre-existing common mental disorders other than PTSD had a somewhat protective effect (HR 0.85, 95% CI 0.76–0.95) against long-term sickness absence (Supplementary Table S3).

### Disability pension

In the crude analyses, the relative risk for being granted disability pension was nearly two times higher among refugees (HR 1.87, 95% CI 1.57–2.23), compared with the Swedish, while other groups had also shown such elevated risks but to a lesser extent, i.e., for non-refugees (HR 1.63, 95% CI 1.50–1.78), and second-generation migrants (HR 1.28, 95% CI 1.15–1.42). The risk estimates attenuated following adjustment for sociodemographic and pre-existing health-related factors, especially among refugees (HR 1.49, 95% CI 1.24–1.77) (Table 4).

**Table 4:** Crude and multivariable hazard ratios (HRs) with 95% confidence intervals (CIs) for *disability pension* among individuals born between 1960 anf 1995, registered as living in Sweden during 2004–2009, age 19 years or older in 2010, with posttraumatic stress disorder diagnosed between 2006 and 2009

Characteristics	No. (%)	HR (95% CI) for disability pension		
		Model 1^a^	Model 2^b^	Model 3^c^
Migration status				
Swedish	951 (3.9)	1.00	1.00	1.00
Refugee migrants	72 (7.0)	1.87 (1.57–2.23)	1.47 (1.23–1.76)	1.49 (1.24–1.77)
Non-refugee migrants	300 (4.7)	1.63 (1.50–1.78)	1.42 (1.29–1.55)	1.42 (1.30–1.56)
2nd-generation migrants	235 (4.8)	1.28 (1.15–1.42)	1.19 (1.08–1.33)	1.18 (1.06–1.31)

^a^Model 1: crude analysis

^b^Model 2: adjusted for sex, household disposable income, and family composition

^c^Model 3: further adjusted for pre-existing major somatic disorders

Supplementary analyses show, that in the fully adjusted model, males were observed at a 41% elevated relative risk for subsequent disability pension than their female peers (Supplementary Table S4). The risk gradually increased with decreasing disposable household income (HR range 0.43–0.78). Furthermore, living with partner and child seemed to reduce the hazards for subsequent disability pension (HR 0.78, 95% CI 0.65–0.93), compared with those living with a partner but no child. Other types of family composition did not seem to have an impact on subsequent disability pension among individuals with PTSD. A significant increase in the likelihood for subsequent disability pension was observed among individuals with PTSD who also had a pre-existing major somatic disorder (HR: 2.16, 95% CI 1.93–2.43), while other pre-existing mental health condition (except PTSD) did not show such association (Supplementary Table S4.).

## Discussion

This longitudinal register-based cohort study among individuals with PTSD aimed to identify the association between migration status and subsequent labour market marginalisation, and to elucidate the impact of the sociodemographic factors and pre-existing health conditions on such association.

### Main findings

The study confirmed that migration status is associated with long-term unemployment, long-term sickness absence, and disability pension respectively among individuals with PTSD. After controlling for several sociodemographic factors and pre-existing health conditions, the risk estimates were attenuated but generally remained higher among refugees, non-refugees and second-generation migrants, compared with the Swedish reference group. The first-generation migrants (i.e., refugees and non-refugees) with PTSD had elevated relative risks for all subsequent labour market marginalisation events, compared with their Swedish peers. The finding is consistent with other Swedish studies suggesting that migrants with poor mental health condition seem more susceptible to labour market marginalisation events than the host population [28, 38].

Refugees were estimated to have the highest likelihood for labour market marginalisation among all migrant groups, compared to the Swedish. However, the difference in the relative risk regarding long-term sickness absence and disability pension comparing within migrant groups was not found statistically significant.

### Long-term unemployment

Migrants are normally considered as a disadvantaged population in relation to acquire and to maintain a job. This disadvantaged situation is even worse among the refugee population in Sweden as many of whom are employed on a temporary basis with lower income [18, 20]. Additionally, the language barrier, acculturation process, and lack of required skills for employment make it more challenging for refugees than other migrants to integrate into the local labour market. The second-generation migrants had a significantly lower risk for long-term unemployment than other groups. This could be due to the second-generation migrants, compared with the foreign-born individuals (i.e. refugees and non-refugees) share many characteristics with the Swedish, in terms of language and culture, and thus have stronger ties to the labour market [30]. Such difference in long-term unemployment rate among those born inside and outside the country has also been reported in the Swedish general population, which is much higher among both refugees and non-refugees (15.1%), than the individuals born in Sweden (4.4%) [20]. The differences between refugees and non-refugees could partly be explained with non-refugees to a large extent being labour market migrants or students, hence they have or get stronger ties with the Swedish labour market. It is not unlikely that refugees have a more complex PTSD than non-refugees, however, we do not have data on the severity of PTSD.

### Long-term sickness absence

In terms of long-term sickness absence, the relative risks among the first-generation migrants (i.e., refugees and non-refugees) were slightly higher than their Swedish peers, however, much lower than that of disability pension or long-term unemployment. According to the Swedish social insurance regulations, one is not eligible for claiming sickness absence benefit if not employed, meaning one on long-term unemployment would not be at risk for long-term sickness absence, therefore it is logical to expect that a high unemployment rate would lead to a low long-term sickness absence in the first-generation migrants. The comparatively low risks in all groups can also be explained by the fact that most people in the labour market had their sickness absence less than 90 net days regardless of their migration status [20, 33].

### Disability pension

The risk estimates for disability pension, even after adjusting for potential sociodemographic and health-related factors, were found high across all migrant groups, especially among the first-generation, while second-generation migrants had only 18% higher relative risk compared to their Swedish peers. These findings are consistent with other Swedish studies, which observed that migrant groups, in general, have a higher risk of disability pension [22, 39, 40]. This might be due to the pre-migration factors, such as inadequate disease prevention in the country of origin, high prevalence of unhealthy behaviour among the migrant population like smoking, obesity, etc. [40, 41].

### Other factors impacting association between migration status and labour market marginalisation

Sociodemographic factors and pre-existing health conditions like major somatic disorders were observed to be influencing the likelihood of labour market marginalisation in individuals with PTSD. We observed an inverse dose–response relationship between household disposable income and risk estimates in all three measures of labour market marginalisation. This is confirming the findings from previous studies that higher disposable income in general populations is identified as a protective factor against labour market marginalisation, and adding to the knowledge that even among persons with a disabling disorder as PTSD income seems protective [27, 42, 43]. This could be explained as either high-income jobs might be easier to maintain while having PTSD, or that the families with higher income are better educated and consequently have a better help-seeking behaviour, resulting in better health to be in the labour market. This again points to the inequalities in health [44]. Males were found to have an elevated risk of disability pension and long-term unemployment compared with females. However, the sex effect was reversed when it came to long-term sickness absence. This difference can be explained by the fact that in Sweden female have in average more days of sick leave than male, and by the uneven sex distribution among the recipients of sickness benefit, where female accounted for more than 60% [33]. Supplementary analyses also showed that pre-existing major somatic disorders was significantly associated with all three outcomes regardless of the migration status, whereas pre-existing psychiatric comorbidity was not. Little or no impact from comorbid psychiatric conditions on labour market marginalisation among individuals with obsessive–compulsive disorder was previously reported from a recent Swedish study [29]. This might partly be because the individuals with psychotic disorders, who carry a high risk of labour market marginalisation [26, 27] were already excluded from the study population, also partly due to the fact that many with other forms of severe psychiatric disorders, or mental retardation, etc., are less likely to join the labour market, thereby the population size was not sufficient to yield significant results.

### Strengths and limitations

The study’s main strength is the use of linked nationwide population registers, which have a comprehensive coverage, good validity of exposures, outcome and other included covariates, and avoids selection bias [45–47]. The outcome measurements are related to the monetary compensation provided by the government, and thereby the quality is further guaranteed [33]. The register-based study design has a very good internal and external validity, and the result could be generalised to other countries where the social structure and welfare system and universal health care system is similar to Sweden[45–47]. Moreover, it is known that individuals with severe mental disorders, e.g., schizophrenia, psychosis, etc. are already at an excessive risk for LMM [26, 27], which hinders their labour market attachment, while people with PTSD or other CMDs have still a potential to engage into the labour market if timely and adequate measures are taken, as these disorders are treatable and likely to worsen with inactivity. However, the majority of the existing research did not exclude/distinguish between psychotic disorders and CMDs including PTSD, neither have they discriminated the risk of labour market marginalisation due to PTSD. The results from our study exclusively singled out the risk factors which impede labour market participation among the population affected by PTSD which in turn help policy makers come up with tailor-made interventions for the group in need. Additionally, the conclusion could also be relevant to other setting to shed light on the risk the PTSD individuals had in terms of labour market marginalisation. Furthermore, the cohort was followed over a long period, namely seven years, with a minimum loss to follow-up, which ensures a well-defined temporality.

Some limitations of the study should be mentioned. The migrants resettling in Sweden in recent years as part of the refugee diaspora from conflicts in Syria, Afghanistan, northern Africa, etc., may be different from our study population, who were living in Sweden during 2006–2009. However, according to the annual statistics from the Swedish Migration Agency, both migration waves (2005–2010 and post-2010) are similar in many ways, including the distribution of migrants’ region of origin, reasons for migration, as well as the approximate sex ratio, except in scales which post-2010 wave is apparently greater [12]. The recent increasing refugee inflow due to political unrest may have resulted in higher PTSD incidences and prevalence among the recently resettled refugee population. Unfortunately, we did not have access to the recent data. Nevertheless, given that our study population included only those with clinically diagnosed PTSD from Swedish National Patient Registers with comprehensive and reliable coverage [46], indicating considerable clinical severity of the underlying PTSD to have been treated at the specialized care, and as the study included a large cohort, we believe that our estimates are not distorted by the possible higher PTSD frequency among the recent refugees other than a slight probable under estimation. Additionally, there were no major changes in the health care or migration-related policy [48, 49], therefore, we consider our results to be generalizable within the Swedish context and to other counties with a similar situation (e.g., other Nordic countries) [50]. Future studies should focus on more recently settled immigrants including refugees and probable differences in the integration process between earlier and recent cohorts of immigrants from both societal and medical perspective. The study included PTSD cases only from inpatient and specialised outpatient diagnose, as the national patient register does not record information on primary care. Given that many PTSD patients are likely to be treated at primary care, the actual number of cases could be underreported, and thereby the actual risk estimates might be underestimated. Besides, the type and severity of PTSD symptom are not covered by the national patient register, which might have impacted the risk estimates as it has been shown that severity is associated with work impairment [51, 52]. Our study could not take into consideration the treatment effect, measured as recovery, remission or recurrence of PTSD, due to lack of data. Such information can be important as the provided treatment may have a differential impact across study subgroups. For instance, PTSD treatment may seem less favourable for migrants due to more complex PTSD, higher co-morbidity, language problems, etc., which in turn may impact subsequent labour market marginalisation. On the other hand, studies show that clinical improvement does not necessarily indicate improvement in work productivity [53]. Nevertheless, future research should include treatment impact when assessing labour market marginalisation in patients with PTSD. Additionally, determining the temporality of pre-existing disorders in relation to PTSD was not in the scope of the available data. As we have mentioned earlier, the national patient register does not include information on treatment, so the temporality in relation to PTSD treatment is not possible to retrieve. However, pre-existing disorders considered in this study are of chronic nature and likely to coexist with PTSD. Nevertheless, determining temporality is a major challenge in psychiatric research. The recorded date in the registers merely reflects the date of treatment seeking and not necessarily the onset of the disorder. Moreover, migration status was the exposure of the study and PTSD was the inclusion criteria. Our study did not aim to identify the consequences of PTSD, but of migration status in patients with PTSD. Therefore, the temporality of pre-existing disorders in relation to PTSD is unlikely to impact our identified association. Lastly, we were unable to retrieve pre-migration factors including health information for the migrants born outside of Sweden, thereby the risk estimates could be subjected to the healthy migrant effect [51].

## Conclusion

The result revealed that among individuals with PTSD, migration status is associated with subsequent long-term unemployment, long-term sickness absence, and disability pension. First-generation migrants, specifically those with a refugee background, are the foremost to be marginalised from the Swedish labour market. The situation with the second-generation migrants in terms of labour market marginalisation, although worse than the host population, yet much better than the first-generation migrants. These findings raise attention among health care workers, employers, and decision-makers. Effective treatment for PTSD is available and should be practiced timely and adequately to prevent early labour market marginalisation. Further research should consider including a more recent migrant population, collecting more detailed clinical information on PTSD severity and pre-migration factors of the migrants in the country of origin by supplementing data from surveys.

## Supplementary Information

Below is the link to the electronic supplementary material.

Supplementary file1 (DOCX 215 KB)

Supplementary file2 (DOCX 33 KB)

## Data Availability

The register data used in this study contain sensitive information at an individual level and therefore, are not publicly available due to confidentiality.

## Code availability

Can be made available upon request.
