# Supplementary figures and images for: Association between migration status and subsequent labour market marginalisation among individuals with posttraumatic stress disorder: a Swedish nationwide register-based cohort study
Source: Soc Psychiatry Psychiatr Epidemiol. 2022 Mar 21;57(5):1073–83. doi: 10.1007/s00127-022-02263-5 (PMC9042996; doi:10.1007/s00127-022-02263-5)

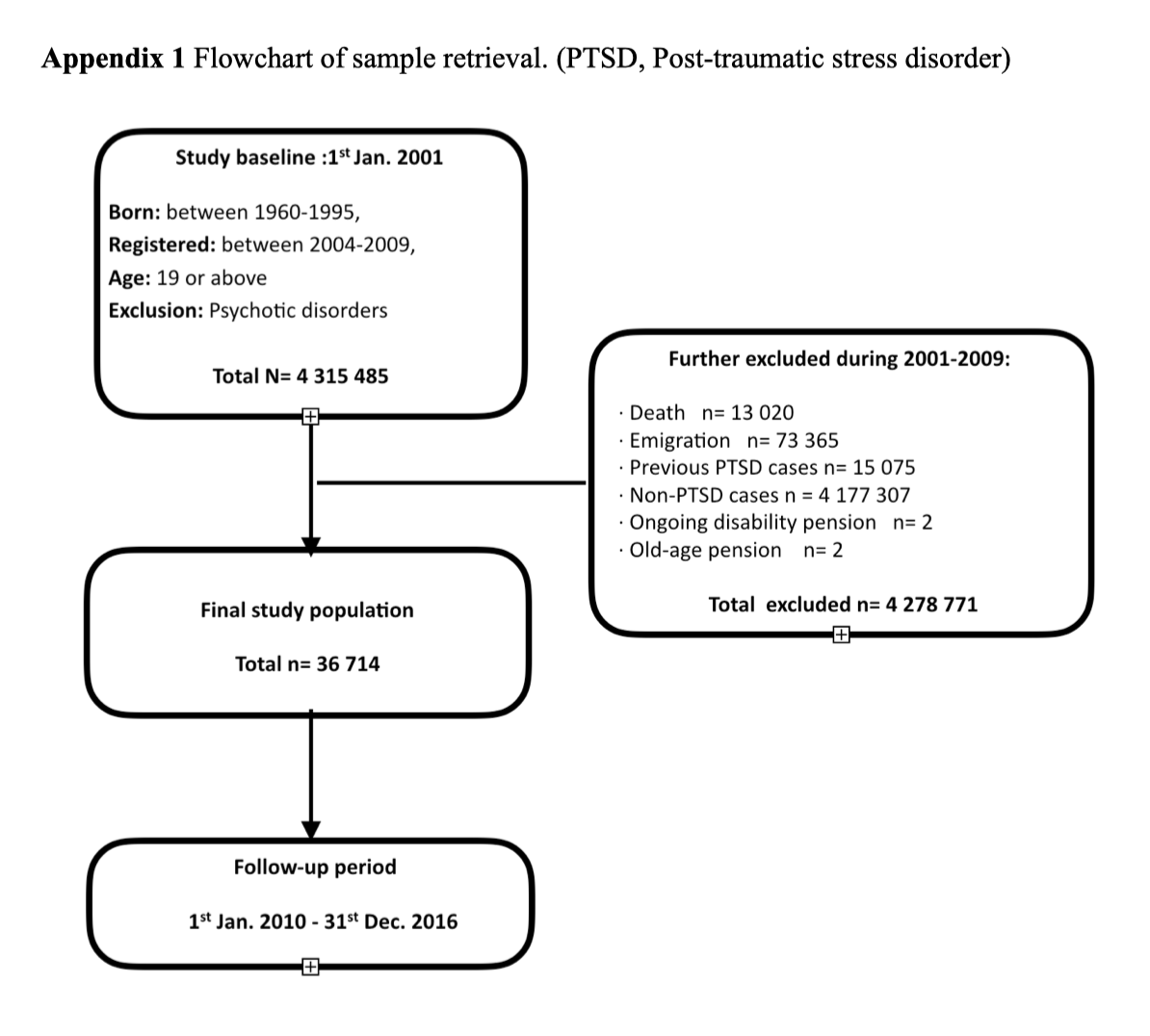

Supplement: Supplementary file 1 — Supplementary file1 (DOCX 215 KB) [file 127_2022_2263_MOESM1_ESM.docx]
