# Supplementary material for: Association between migration status and subsequent labour market marginalisation among individuals with posttraumatic stress disorder: a Swedish nationwide register-based cohort study
Source: Soc Psychiatry Psychiatr Epidemiol. 2022 Mar 21;57(5):1073–83. doi: 10.1007/s00127-022-02263-5 (PMC9042996; doi:10.1007/s00127-022-02263-5)
Supplement: Supplementary file 2 — Supplementary file2 (DOCX 33 KB) [file 127_2022_2263_MOESM2_ESM.docx]

| **Table S1.** Diagnostics with corresponding ICD-10 codes used in the study | |
| --- | --- |
| **Disease** | **ICD10 Codes** |
| Posttraumatic stress disorder (PTSD) | F43.1 |
| Major somatic disorders  - Circulatory system diseases | I00-I99 |
| - Respiratory system diseases | J00-J99 |
| - Diabetes mellitus | E10-E14 |
| - Musculoskeletal system diseases | M00-M99 |
| Other common mental disorders  - Depressive episode | F32-F33 |
| - Anxiety disorders other than PTSD | F40-F43 (Except F43.1) |
| Other mental disorders | All ICD-10: F codes other than above mentioned |

**Supplementary Tables**

| **Table S2.** Crude and multivariable hazard ratios (HRs) with 95 % confidence intervals (CIs) for long-term unemployment (>180 days) among individuals born between 1960-1995, registered as living in Sweden in 2004-09, age 19 years or older in 2010, with posttraumatic stress disorder diagnosed between 2006-09 ^a^ | | | | |
| --- | --- | --- | --- | --- |
|  | Cases (%) | Model 1^b^ | Model 2^c^ | Model 3^d^ |
|  |  | HR (95% CI) | | |
| *Sociodemographic factors* |  |  |  |  |
| *Migration Status* |  |  |  |  |
| Swedish | 2 177 (8.9) | 1 | 1 | 1 |
| Refugee Migrants | 286 (28.0) | 2.81 (2.53-3.12) | 2.06 (1.86-2.30) | 2.07 (1.86-2.30) |
| Non-refugee Migrants | 1 540 (24.2) | 2.42 (2.29-2.55) | 1.95 (1.84-2.06) | 1.96 (1.85-2.07) |
| 2^nd^-generation Migrants | 617 (12.7) | 1.38 (1.29-1.48) | 1.28 (1.19-1.37) | 1.27 (1.18-1.36) |
| *Sex* |  |  |  |  |
| Female | 2 679 (59.3) | 1 | 1 | 1 |
| Male | 1 842 (40.7) | 1.18 (1.13-1.24) | 1.25 (1.19-1.31) | 1.27 (1.21-1.33) |
| *Age (years)* |  |  |  |  |
| 19-29 | 1 004 (22.2) | 1 | 1 | 1 |
| 30 or more | 3 517 (77.8) | 0.95 (0.90-0.99) | 0.91 (0.84-0.98) | 0.88 (0.81-0.95) |
| *Weighted household disposable income (in quintile)* | | | | |
| Lowest | 2 074 (45.9) | 1 | 1 | 1 |
| Second | 867 (19.2) | 0.55 (0.52-0.59) | 0.63 (0.59-0.67) | 0.63 (0.59-0.67) |
| Third | 683 (15.1) | 0.43 (0.40-0.46) | 0.51 (0.48-0.55) | 0.52 (0.48-0.55) |
| Fourth | 553 (12.2) | 0.37 (0.34-0.40) | 0.43 (0.40-0.46) | 0.43 (0.40-0.47) |
| Highest | 344 (7.6) | 0.31 (0.28-0.34) | 0.34 (0.31-0.38) | 0.35 (0.32-0.39) |
| *Family composition* |  |  |  |  |
| Living with partner without children | 287 (6.3) | 1 | 1 | 1 |
| Living with partner and children | 1 409 (31.2) | 0.66 (0.59-0.73) | 0.72 (0.64-0.8) | 0.71 (0.64-0.79) |
| Single without children | 1 844 (40.8) | 0.80 (0.73-0.89) | 0.98 (0.88-1.09) | 0.98 (0.88-1.08) |
| Single with children | 548 (12.1) | 0.98 (0.87-1.11) | 1.02 (0.91-1.15) | 1.01 (0.89-1.13) |
| Single age < 20, living with parents | 433 (9.6) | 0.67 (0.60-0.76) | 0.7 (0.61-0.8) | 0.68 (0.59-0.78) |
| *Pre-existing health condition* ^e, f^ |  |  |  |  |
| *Major somatic disorders* |  |  |  |  |
| No | 812 (18.0) | 1 |  | 1 |
| Yes | 3 709 (82.0) | 1.42 (1.33-1.51) |  | 1.40 (1.32-1.5) |
| *Other common mental disorders* |  |  |  |  |
| No | 4 244 (93.9) | 1 |  |  |
| Yes | 277 (6.1) | 0.94 (0.86-1.04) |  |  |
| *Other mental disorders* |  |  |  |  |
| No | 4 361 (96.5) | 1 |  |  |
| Yes | 160 (3.5) | 1.11 (0.98-1.27) |  |  |
| ^a^ All variables were measured at the end of 2009.  ^b^ Model 1: Crude analysis.  ^c^ Model 2: Adjusted for age, sex, household disposable income, and family composition.  ^d^ Model 3: Further adjusted for previous major somatic disorders  ^e^ Pre-existing refers to before the follow-up (1-Jan-2010)  ^f^ For detailed disease classification please see Supplementary Table S1. | | | | |

| **Table S3.** Crude and multivariable hazard ratios (HRs) with 95 % confidence intervals (CIs) for long-term sickness absence (>90 net days) among individuals born between 1960-95, registered as living in Sweden in 2004-09, age 19 years or older in 2010, with posttraumatic stress disorder diagnosed between 2006-09^a^ | | | | |
| --- | --- | --- | --- | --- |
|  | Cases (%) | Model 1^b^ | Model 2^c^ | Model 3^d^ |
|  |  | HR (95% CI) | | |
| *Sociodemographic factors* |  |  |  |  |
| *Migration Status* |  |  |  |  |
| Swedish | 2 870 (11.7) | 1 | 1 | 1 |
| Refugee Migrants | 114 (11.1) | 1.30 (1.14-1.48) | 1.16 (1.02-1.33) | 1.18 (1.03-1.35) |
| Non-refugee Migrants | 678 (10.6) | 1.21 (1.14-1.28) | 1.10 (1.03-1.17) | 1.11 (1.04-1.19) |
| 2^nd^-generation Migrants | 616 (12.6) | 1.16 (1.09-1.24) | 1.14 (1.06-1.22) | 1.13 (1.05-1.21) |
| *Sex* |  |  |  |  |
| Female | 3 003 (70.9) | 1 | 1 | 1 |
| Male | 1 231 (29.1) | 0.88 (0.84-0.93) | 0.90 (0.86-0.95) | 0.92 (0.88-0.97) |
| *Age (years)* |  |  |  |  |
| 19-29 | 932 (22.0) | 1 | 1 | 1 |
| 30 or more | 3 302 (78.0) | 0.94 (0.89-0.99) | 0.98 (0.92-1.03) | 0.94 (0.89-0.99) |
| *Weighted household disposable income (in quintile)* | | | | |
| Lowest | 1 096 (25.9) | 1 | 1 | 1 |
| Second | 926 (21.9) | 0.87 (0.81-0.92) | 0.89 (0.83-0.95) | 0.88 (0.83-0.95) |
| Third | 929 (21.9) | 0.78 (0.73-0.83) | 0.80 (0.75-0.86) | 0.81 (0.76-0.87) |
| Fourth | 786 (18.6) | 0.70 (0.66-0.76) | 0.73 (0.68-0.79) | 0.74 (0.69-0.80) |
| Highest | 497 (11.7) | 0.58 (0.53-0.63) | 0.60 (0.55-0.66) | 0.62 (0.57-0.67) |
| *Family composition* |  |  |  |  |
| Living with partner without children | 173 (4.1) | 1 |  |  |
| Living with partner and children | 1 465 (34.6) | 0.91 (0.81-1.03) |  |  |
| Single without children | 1 545 (36.5) | 0.99 (0.88-1.12) |  |  |
| Single with children | 527 (12.4) | 1.28 (1.12-1.46) |  |  |
| Single age < 20, living with parents | 524 (12.4) | 1.03 (0.91-1.18) |  |  |
| *Pre-existing health condition* ^e, f^ |  |  |  |  |
| *Major somatic disorders* |  |  |  |  |
| No | 656 (15.5) | 1 |  | 1 |
| Yes | 3 578 (84.5) | 1.64 (1.53-1.76) |  | 1.60 (1.49-1.71) |
| *Other common mental disorders* |  |  |  |  |
| No | 4 042 (95.5) | 1 |  | 1 |
| Yes | 192 (4.5) | 0.79 (0.71-0.88) |  | 0.85 (0.76-0.95) |
| *Other mental disorders* |  |  |  |  |
| No | 4 119 (97.3) | 1 |  |  |
| Yes | 115 (2.7) | 0.96 (0.84-1.11) |  |  |
| ^a^ All variables were measured at the end of 2009, except pre-existing health condition  ^b^ Model 1: Crude analysis.  ^c^ Model 2: Adjusted for age, sex, household disposable income.  ^d^ Model 3: Further adjusted for previous major somatic disorders, and other common mental disorders.  ^e^ Pre-existing refers to before the follow-up (1-Jan-2010)  ^f^ For detailed disease classification please see Supplementary Table S1. | | | | |

| **Table S4.** Crude and multivariable hazard ratios (HRs) with 95 % confidence intervals (CIs) for disability pension among individuals born between 1960-95, registered as living in Sweden during 2004-09, age 19 years or older in 2010, with posttraumatic stress disorder diagnosed between 2006-09 ^a^ | | | | |
| --- | --- | --- | --- | --- |
|  | Cases (%) | Model 1^b^ | Model 2^c^ | Model 3^d^ |
|  |  | HR (95% CI) | | |
| *Sociodemographic factors* |  |  |  |  |
| *Migration Status* |  |  |  |  |
| Swedish | 951 (3.9) | 1 | 1 | 1 |
| Refugee Migrants | 72 (7.0) | 1.87 (1.57-2.23) | 1.47 (1.23-1.76) | 1.49 (1.24-1.77) |
| Non-refugee Migrants | 300 (4.7) | 1.63 (1.50-1.78) | 1.42 (1.29-1.55) | 1.42 (1.30-1.56) |
| 2^nd^-generation Migrants | 235 (4.8) | 1.28 (1.15-1.42) | 1.19 (1.08-1.33) | 1.18 (1.06-1.31) |
| *Sex* |  |  |  |  |
| Female | 937 (60.3) | 1 | 1 | 1 |
| Male | 618 (39.7) | 1.33 (1.24-1.42) | 1.38 (1.29-1.49) | 1.41 (1.31-1.52) |
| *Age (years)* |  |  |  |  |
| 19-29 | 330 (21.2) | 1 |  |  |
| 30 or more | 1 225 (78.8) | 0.94 (0.87-1.02) |  |  |
| *Weighted household disposable income (in quintile)* | | | | |
| Lowest | 592 (38.1) | 1 | 1 | 1 |
| Second | 376 (24.2) | 0.73 (0.67-0.81) | 0.79 (0.72-0.87) | 0.78 (0.71-0.86) |
| Third | 309 (19.9) | 0.57 (0.52-0.64) | 0.63 (0.57-0.70) | 0.64 (0.58-0.71) |
| Fourth | 175 (11.3) | 0.44 (0.39-0.49) | 0.47 (0.42-0.53) | 0.48 (0.42-0.54) |
| Highest | 103 (6.6) | 0.40 (0.35-0.46) | 0.41 (0.36-0.48) | 0.43 (0.37-0.49) |
| *Family composition* |  |  |  |  |
| Living with partner without children | 45 (2.9) | 1 | 1 | 1 |
| Living with partner and children | 530 (34.1) | 0.79 (0.67-0.95) | 0.79 (0.66-0.94) | 0.78 (0.65-0.93) |
| Single without children | 536 (34.5) | 1.00 (0.85-1.19) | 1.08 (0.90-1.28) | 1.08 (0.90-1.28) |
| Single with children | 210 (13.5) | 1.14 (0.94-1.39) | 1.15 (0.94-1.40) | 1.10 (0.90-1.34) |
| Single age < 20, living with parents | 234 (15.0) | 1.05 (0.87-1.27) | 1.03 (0.86-1.30) | 1.06 (0.87-1.28) |
| *Pre-existing health condition* ^e, f^ |  |  |  |  |
| *Major somatic disorders* |  |  |  |  |
| No | 92 (5.9) | 1 |  | 1 |
| Yes | 1 463 (94.1) | 2.16 (1.92-2.42) |  | 2.16 (1.93-2.43) |
| *Other common mental disorders* |  |  |  |  |
| No | 1 485 (95.5) | 1 |  |  |
| Yes | 70 (4.5) | 0.86 (0.74-1.01) |  |  |
| *Other mental disorders* |  |  |  |  |
| No | 1 512 (97.2) | 1 |  |  |
| Yes | 43 (2.8) | 0.97 (0.79-1.20) |  |  |
| ^a^ All variables were measured at the end of 2009, except pre-existing health condition  ^b^ Model 1: Crude analysis.  ^c^ Model 2: Adjusted for age, sex, household disposable income, and family composition.  ^d^ Model 3: Further adjusted for previous major somatic disorders.  ^e^ Pre-existing refers to before the follow-up (1-Jan-2010)  ^f^ For detailed disease classification please see Supplementary Table S1. | | | | |

**Table S5.** Adjusted hazard ratios (aHRs) with 95% confidence intervals (CIs) for Disability pension among all individuals in the study base

| Population categories | aHRs | CIs |
| --- | --- | --- |
| Swedish without PTSD (reference) | 1 | - |
| Swedish with PTSD | 1.98 | (1.89-2.07) |
| 2^nd^ generation migrants without PTSD | 1.39 | (1.38-1.41) |
| 2^nd^ generation migrants with PTSD | 2.34 | (2.13-2.57) |
| Non-refugee migrant without PTSD | 2.14 | (2.11-2.17) |
| Non-refugee migrant with PTSD | 2.47 | (2.30-2.66) |
| Refugee migrant without PTSD | 1.47 | (1.44-1.51) |
| Refugee migrant with PTSD | 2.56 | (2.16-3.03) |

**Table S6.** Adjusted hazard ratios (aHRs) with 95% confidence intervals (CIs) for long-term sickness absence among all individuals in the study base

| Population categories | aHRs | CIs |
| --- | --- | --- |
| Swedish without PTSD (reference) | 1 | - |
| Swedish with PTSD | 1.45 | (1.41-1.49) |
| 2^nd^ generation migrants without PTSD | 1.16 | (1.15-1.17) |
| 2^nd^ generation migrants with PTSD | 1.63 | (1.53-1.73) |
| Non-refugee migrant without PTSD | 1.69 | (1.68-1.70) |
| Non-refugee migrant with PTSD | 1.53 | (1.45-1.61) |
| Refugee migrant without PTSD | 1.17 | (1.15-1.19) |
| Refugee migrant with PTSD | 1.64 | (1.44-1.87) |

**Table S7.** Hazard ratios (HRs) with 95% confidence intervals (CIs) for long-term unemployment among all individuals in the study base

| Population categories | aHRs | CIs |
| --- | --- | --- |
| Swedish without PTSD (reference) | 1 | - |
| Swedish with PTSD | 1.39 | (1.35-1.44) |
| 2^nd^ generation migrants without PTSD | 1.37 | (1.36-1.38) |
| 2^nd^ generation migrants with PTSD | 1.75 | (1.65-1.86) |
| Non-refugee migrant without PTSD | 2.48 | (2.47-2.50) |
| Non-refugee migrant with PTSD | 2.64 | (2.53-2.75) |
| Refugee migrant without PTSD | 2.67 | (2.63-2.70) |
| Refugee migrant with PTSD | 2.82 | (2.55-3.11) |
